# Proper management of rheumatoid arthritis in Latin America. What the guidelines say?

**DOI:** 10.1007/s10067-015-3016-9

**Published:** 2015-07-23

**Authors:** Claiton V. Brenol, Jorge Ivan Gamez Nava, Enrique R. Soriano

**Affiliations:** Rheumatology Unit, Hospital de Clinicas de Porto Alegre, Rio Grande do Sul, Brazil; Faculdade de Medicina, Universidade Federal do Rio Grande do Sul, Rio Grande do Sul, Brazil; Unidad de Investigación en Epidemiologia Clínica, UMAE Hospital de Especialidades del Centro Médico Nacional de Occidente, Instituto Mexicano del Seguro Social, Guadalara, Mexico; Programas de Doctorado en Salud Publica y Farmacologia, Centro Universitario de Ciencias de la Salud, Universidad de Guadalajara, Guadalajara, Mexico; Rheumatology Unit, Internal Medicine Service, Hospital Italiano de Buenos Aires, Peron 4190, (1180), Buenos Aires, CABA Argentina; Instituto Universitario, Hospital Italiano de Buenos Aires, Peron 4190, (1180), Buenos Aires, CABA Argentina

**Keywords:** Clinical guidelines, Treatment rheumatoid arthritis

## Abstract

To analyze characteristics of clinical practice guidelines (CPGs) for the management of rheumatoid arthritis (RA) developed in Latin American (LA) countries and to describe the knowledge, use, and barriers for their implementation perceived among LA rheumatologists, a comprehensive literature search including Medline, PubMed, Cochrane Library, LILACS and Scielo was performed. The Appraisal of Guidelines for Research and Evaluation (AGREE) instrument was applied for evaluation. A survey was sent to PANLAR members containing questions related to knowledge about guidelines, application of the recommendations, and difficulties in implementing CPGs. Eight guidelines were identified. Most guidelines were evidence based (62 %), but in only 37 % a systematic literature search was done. None of the guidelines included patients’ views and preferences, and only few of them stated an updating procedure. Funding body independence and disclosure of conflicts of interest were rarely reported. The survey was answered by 214 rheumatologists from all Latin American countries. Most rheumatologist reported knowledge and use of clinical guidelines, mainly international ones. In general, rheumatologist felt that guidelines apply to only a minority of patients seen in daily clinical practice. Limited access expensive drugs, suggested by the guidelines, was the most frequent barrier to guidelines implementation that was reported. A good number of guidelines on the treatment of rheumatoid arthritis have been developed in Latin America. Most of them are lacking some of the components recognized for high-quality clinical guidelines development. In spite that most rheumatologist know and apply guidelines, access to drugs is still a very important barrier to their implementation in Latin America.

## Introduction

The movement to develop and disseminate clinical practice guidelines (CPGs) has been well established for more than two decades [1]. This movement was initially rooted somewhat in the need to curtail or restrict practice variation in the US health-care system and slowly widespread to the rest of the world, very much linked to the evidence-based medicine movement [1, 2].

As the years passed the procedures and requirements for development of clinical guidelines have been more and more standardized, guideline clearing houses created, and robust guideline appraisal instruments developed [3–6].

Guidelines are not self-implementing. Developing guidelines and making them available to health-care professionals does not ensure their use [4]. Two of the key issues related to the success in the implementation of clinical guidelines are the quality of the guideline and that it is developed or approved by a credible body or association [1].

In 2014, the European League Against Rheumatism (EULAR) updated the standardized operating procedures for the elaboration, evaluation, dissemination, and implementation of recommendations endorsed by the EULAR standing committees for musculoskeletal disorders, either in general (e.g., inflammatory diseases) or specific (e.g., rheumatoid arthritis) [7, 8]. EULAR states that for successful implementation, a separate project is usually necessary, selecting one or more established implementation strategies, such as knowledge about the recommendations, inclusion of recommendations in quality indicators, reimbursement dependent on fulfillment of certain recommendations, and audit feedback [7].

Several guidelines or recommendations have been developed in Latin America for the management of rheumatoid arthritis. The aim of this study is to describe and analyze the characteristics and differences of these guidelines, and to describe a survey performed among Latin American rheumatologists, related to the knowledge and use of clinical guidelines for the management of rheumatoid arthritis, and the implementation of clinical guidelines in their own setting.

## Methods

A comprehensive literature search was performed including the following databases: Medline, PubMed, Cochrane Library, LILACS, and Scielo. The words rheumatoid arthritis, and recommendation or guide or guidelines and Latin America, or each one of the Latin American countries was used for the search. All Latin American rheumatology societies or associations were contacted, and their official journals were searched by hand looking for rheumatoid arthritis guidelines or recommendations. Only guidelines developed by scientific societies have been included; recommendations or guidelines developed by governments or regulatory agencies were not considered. When a guideline had several updates, only the last one was included.

Guidelines were analyzed using the Appraisal of Guidelines for Research and Evaluation (AGREE) instrument [6] and summarized in a table.

On the other hand, a survey with five specific questions related to the use of treatment guidelines in clinical practice was sent to all PANLAR members. The main topics addressed were knowledge about guidelines worldwide, application of the recommendations, extrapolation to whole rheumatoid arthritis (RA) patient population, and difficulties in implementing clinical guidelines in different countries.

## Results

Nine National and one Latin American guidelines (Table 1) were identified [9–16] (Three out of 35 papers retrieved were identified in PubMed: Mexican, Brazilian, and PANLAR; two extra ones out of 40 papers retrieved were identified in LILACS-Scielo: Argentinean and Chilean; No results were obtained from the Cochrane database; another five guidelines were found in non-indexed Societies Journals or after asking the Societies). Guideline characteristics according to the AGREE instrument [6] are summarized in Table 1. Six of the 10 (60 %) guidelines were evidence based, and in four (40 %) a systematic literature review was performed. None of the guidelines included patients’ views and preferences. Only three guidelines (30 %) provided a procedure for updating the guideline, four (40 %) were editorially independent from the funding body, and in two conflicts of interest of guideline development members were recorded. Only one guideline considered potential cost implications of applying the recommendations.

**Table 1:** Guidelines characteristics according to some of components of the AGREE instrument [6]

		Scope and purpose	Stakeholder involvement	Rigor of development			Clarity and presentation	Applicability	Editorial independence	
Country	Year	Overall objective(s) of the guideline is (are) specifically described	Patients’ views and preferences included	Evidence based	Systematic literature search done	Updating procedure provided	Recommendations specific and unambiguous	Cost implications considered	Editorially independent from the funding body	Conflicts of interest recorded
Colombia [10]	2002	No	No	Yes	No	No	Yes	No	No	No
PANLAR [9]	2006	Yes	No	Yes	No	No	Yes	No	Yes	No
Uruguay	2007	Yes	No	No	No	No	Yes	No	Yes	No
Chile [16]	2007	Yes	No	No	No	No	Yes	No	No	No
Ecuador	2010	Yes	No	No	No	No	Yes	No	Yes	No
Costa Rica [14]	2011	No	No	No	No	No	No	No	No	No
Venezuela	2011	Yes	No	Yes	Yes	No	Yes	No	No	No
Brazil [19]	2012	Yes	No	Yes	Yes	Yes	Yes	Yes	Yes	Yes
Argentina [15]	2013	Yes	No	Yes	Yes	Yes	Yes	Yes	No	No
Mexico [11]	2014	Yes	No	Yes	Yes	Yes	Yes	No	No	Yes

All guidelines recommended traditional disease modifying antirheumatic drugs (DMARDs) monotherapy or DMARDs combination as first line therapy (Table 2). All guidelines suggested failure to at least two DMARDs before biologic therapy, except the two newest ones (Mexico and Argentina) that accepted biologics after failure to just one traditional DMARD. None of the guidelines recommended biologics or small molecules as first-line therapy.

**Table 2:** Summary of Latin American guidelines recommendations

Country	First line treatment	Number of DMARDs suggested before biologics	Combination of DMARDs suggested before biologics	Biologics considered as first line	Small molecules recommended
Colombia [10]	DMARDs	Not stated	Yes	No	NA
PANLAR [9]	DMARDs	Two (including combination DMARDs)	Yes	No	NA
Uruguay	DMARDs	Two	Yes	No	NA
Chile [16]	DMARDs	Two	Yes	No	NA
Ecuador	DMARDs	Two	Yes	No	NA
Costa Rica [14]	DMARDs	Two	Yes	No	NA
Venezuela	DMARDs	Two	Yes	No	NA
Brazil [19]	DMARDs	Two	Yes	No	NA
Argentina [15]	DMARDs	One	Yes	Yes (Patients with contraindications for DMARDs)	After biologics
Mexico [11]	DMARDs	One	Yes	No	After biologics

*DMARDs* disease modifying antirheumatic drugs, *NA* not applicable

Two hundred and fourteen rheumatologists, from different Latin American (LA) countries, answered the survey. Most of the rheumatologists reported knowledge about at least one guideline on the treatment of RA (94.59 %). Among them, 82.45 % declared that they apply the recommendations in their daily clinical practice. Several guidelines from different countries and scientific societies were mentioned as used for treating patients with RA. Guidelines from the American College of Rheumatology (ACR) and European League Against Rheumatism (EULAR) were reported as the more frequently used. However, recommendations of other scientific societies were cited, such as from Argentina, Brazil, Bolivia, Chile, Colombia, Costa Rica, Mexico, and Spain. Some countries have government guidelines that are used by rheumatologists like Brazil, Chile, and Peru. More than a half of physicians answering the survey referred that guidelines are not suitable for a high proportion of their patients, and are not suitable and practical for the management of individual patient. According to their view, this was due at least in part, to the presence of comorbidities in their population, not contemplated in the guidelines.

An interesting point about barriers to implementation of guidelines in clinical settings was addressed. Most of the physicians (63.9 %) think that patients have limited access to drugs. Several reasons were mentioned to explain that, including poorly organized health-care systems, government economic issues, low socioeconomic status, and low education level. Another explanation suggested is that there might be a lack of knowledge about the guidelines by physicians treating patients with RA. A low percentage of responders had difficulties using the guidelines because some of them are outdated. Less than 5 % of colleagues do not agree with the treatment recommendations in the guidelines.

## Discussion

In 1992, the Institute of Medicine report defined guidelines as “systematically developed statements to assist practitioner and patient decisions about appropriate health care for specific clinical circumstances.” It is a generalized view that in spite of the existence of internationally accepted guidelines, local guidelines have an important role in the management of diseases at regional level. Several Latin American rheumatology societies and associations members of PANLAR have developed their own guidelines or recommendations for the management of rheumatoid arthritis. Rheumatoid arthritis is a frequent disease in Latin America, and the appearance of new effective high-cost therapies urged medical associations to develop guidelines to standardize the management of this disease, before it was done by governments or payers, which are not directly dealing with patients themselves. As most of the guidelines have been evidence based, they look very much alike.

There are however some issues nowadays considered an indispensable part of high-quality developed clinical practice guidelines, which most of the Latin American guidelines have not included: patients’ views and preferences, procedures for updating the guidelines, and disclosure of conflicts of interest of guideline development members. These issues should be considered in future guidelines to be developed. The survey, although answered by a low number of rheumatologists, produced some interesting results that are very much in line with the previous thoughts on the issue. Most of rheumatologists are aware of the existence of international and local guidelines and they use them in clinical practice. In spite that most of them agree with the guidelines and only a very few minority disagrees, most of them think that the recommendations do not apply to a large number of their patients. That most probably happens because guidelines are evidence based, and the best evidence comes from randomized clinical trials that in fact do exclude many of the usual patients seen in daily clinical practice. The Latin American social economic situation was also pointed out, where in many regions and countries most patients do not have access to effective, high-cost treatments. Inequities among different countries and among different health-care systems even in the same country are the norm in Latin America, and medical associations have an important role to help to narrow the gap.

Guidelines are also important for health authorities and payers, to unify the way patients are treated. In spite that, as shown here, many rheumatology societies have developed their own guidelines, health regulatory authorities do not always accept them, and there is an increased tendency from governments in Latin America to develop their own recommendations. Examples are the Peruvian and Colombian governments [17, 18]. It would be important that these initiatives include the local societies in the elaboration of the guidelines and not just some rheumatologists, to give better representativeness and acceptance by rheumatologists to the recommendation. Even though health authorities and regulatory agencies might try to impose treatment strategies, in most of Latin American laws the ultimate responsible of the prescription is the physician that should be convinced and agree with the indication.

In summary, there is an important work that has been done in Latin America in order to improve the knowledge and management of rheumatoid arthritis according to evidence. There is still however much more to do if we want that the guidelines are fully implemented and patients have access to the treatments they deserve.

## References

[1] Davis DA, Taylor-Vaisey A (1997). Translating guidelines into practice. A systematic review of theoretic concepts, practical experience and research evidence in the adoption of clinical practice guidelines. CMAJ : Canadian Medical Association journal = journal de l’Association medicale canadienne.

[2] Oxman AD, Sackett DL, Guyatt GH (1993). Users’ guides to the medical literature. I. How to get started. The Evidence-Based Medicine Working Group. Jama.

[3] Eccles MP, Grimshaw JM, Shekelle P, Schunemann HJ, Woolf S (2012). Developing clinical practice guidelines: target audiences, identifying topics for guidelines, guideline group composition and functioning and conflicts of interest. Implementation Science: IS.

[4] Shekelle P, Woolf S, Grimshaw JM, Schunemann HJ, Eccles MP (2012). Developing clinical practice guidelines: reviewing, reporting, and publishing guidelines; updating guidelines; and the emerging issues of enhancing guideline implementability and accounting for comorbid conditions in guideline development. Implementation Science: IS.

[5] Woolf S, Schunemann HJ, Eccles MP, Grimshaw JM, Shekelle P (2012). Developing clinical practice guidelines: types of evidence and outcomes; values and economics, synthesis, grading, and presentation and deriving recommendations. Implementation Science: IS.

[6] Collaboration A (2003). Development and validation of an international appraisal instrument for assessing the quality of clinical practice guidelines: the AGREE project. Quality & Safety in Health Care.

[7] van der Heijde D, Aletaha D, Carmona L, Edwards CJ, Kvien TK, Kouloumas M, Machado P, Oliver S, de Wit M, Dougados M (2015). 2014 Update of the EULAR standardised operating procedures for EULAR-endorsed recommendations. Annals of the Rheumatic Diseases.

[8] Dougados M, Betteridge N, Burmester GR, Euller-Ziegler L, Guillemin F, Hirvonen J, Lloyd J, Ozen S, Da Silva JA, Emery P, Kalden JR, Kvien T, Matucci-Cerinic M, Smolen J, Eular (2004). EULAR standardised operating procedures for the elaboration, evaluation, dissemination, and implementation of recommendations endorsed by the EULAR standing committees. Annals of the Rheumatic Diseases.

[9] Cardiel MH, Latin American Rheumatology Associations of the Pan-American League of Associations for R, Grupo Latinoamericano de Estudio de Artritis R (2006). First Latin American position paper on the pharmacological treatment of rheumatoid arthritis. Rheumatology.

[10] Caballero-Uribe CC, P.; Londoño J. (2002) Guías Colombianas para el Tratamiento de la Artritis Reumatoide. Exlibris Editores S. A

[11] Cardiel MH, Diaz-Borjon A, del Mercado V, Espinosa M, Gamez-Nava JI, Barile Fabris LA, Pacheco Tena C, Silveira Torre LH, Pascual Ramos V, Goycochea Robles MV, Aguilar Arreola JE, Gonzalez Diaz V, Alvarez Nemegyei J, Gonzalez-Lopez Ldel C, Salazar Paramo M, Portela Hernandez M, Castro Colin Z, Xibille Friedman DX, Alvarez Hernandez E, Casasola Vargas J, Cortes Hernandez M, Flores-Alvarado DE, Martinez Martinez LA, Vega-Morales D, Flores-Suarez LF, Medrano Ramirez G, Barrera Cruz A, Garcia Gonzalez A, Lopez Lopez SM, Rosete Reyes A, Espinosa Morales R (2014). Update of the Mexican College of Rheumatology guidelines for the pharmacologic treatment of rheumatoid arthritis. Reumatologia Clinica.

[12] da Mota LM, Cruz BA, Brenol CV, Pereira IA, Rezende-Fronza LS, Bertolo MB, de Freitas MV, da Silva NA, Louzada-Junior P, Giorgi RD, Lima RA, da Rocha Castelar Pinheiro G, Brazilian Society of R (2012). 2012 Brazilian Society of Rheumatology Consensus for the treatment of rheumatoid arthritis. Revista Brasileira de Reumatologia.

[13] Soriano ER, Galarza-Maldonado C, Cardiel MH, Pons-Estel BA, Massardo L, Caballero-Uribe CV, Achurra-Castillo AF, Barile-Fabris LA, Chavez-Corrales J, Diaz-Coto JF, Esteva-Spinetti MH, Guibert-Toledano M, Palazuelos FI, Keiserman MW, Lomonte AV, Mota LM, Pineda Villasenor C, Alarcon GS, Gladar (2008). Use of rituximab for the treatment of rheumatoid arthritis: the Latin American context. Rheumatology.

[14] Reumatología ACd (2011). Guides management of rheumatoid arthritis: consensus 2010. Acta Med Costarric.

[15] Reumatología SAd (2013). Update of clinical practice guidelines in rheumatoid arthritis treatment. Rev Argent Reumatol.

[16] Reumatología SCd (2004). Guides of treatment of rheumatoid arthritis. Reumatología (Santiago de Chile).

[17] Guias de Practica Clinica Artritis Reumatoidea. www.essalud.gob.pe/transparencia/pdf/…/guia_artritisreumatoide2011.pd. Accessed 24/04/2015 2015

[18] http://gpc.minsalud.gov.co/guias/Documents/Artritis%20Reumatoidea/GPC%20AR%20COMPLETA.pdf. Accessed 24/04/2015 2015

[19] Mota LM, Cruz BA, Brenol CV, Pereira IA, Rezende-Fronza LS, Bertolo MB, Freitas MV, Silva NA, Louzada-Junior P, Giorgi RD, Lima RA, Bernardo WM, Pinheiro Gda R, Sociedade Brasileira de R (2013). Guidelines for the drug treatment of rheumatoid arthritis. Revista Brasileira de Reumatologia.

